# A comprehensive proteomic approach to identifying capacitation related proteins in boar spermatozoa

**DOI:** 10.1186/1471-2164-15-897

**Published:** 2014-10-14

**Authors:** Woo-Sung Kwon, Md Saidur Rahman, June-Sub Lee, Jin Kim, Sung-Jae Yoon, Yoo-Jin Park, Young-Ah You, Seongsoo Hwang, Myung-Geol Pang

**Affiliations:** Department of Animal Science & Technology, Chung-Ang University, Anseong, Gyeonggi-do, 456-756 Republic of Korea; Animal Biotechnology Division, National Institute of Animal Science, RDA, Suwon, Gyeonggi-do, 441-706 Republic of Korea

**Keywords:** Capacitation, Proteomics, Boar, Spermatozoa, 2DE

## Abstract

**Background:**

Mammalian spermatozoa must undergo capacitation, before becoming competent for fertilization. Despite its importance, the fundamental molecular mechanisms of capacitation are poorly understood. Therefore, in this study, we applied a proteomic approach for identifying capacitation-related proteins in boar spermatozoa in order to elucidate the events more precisely. 2-DE gels were generated from spermatozoa samples in before- and after-capacitation. To validate the 2-DE results, Western blotting and immunocytochemistry were performed with 2 commercially available antibodies. Additionally, the protein-related signaling pathways among identified proteins were detected using Pathway Studio 9.0.

**Result:**

We identified Ras-related protein Rab-2, Phospholipid hydroperoxide glutathione peroxidase (PHGPx) and Mitochondrial pyruvate dehydrogenase E1 component subunit beta (PDHB) that were enriched before-capacitation, and NADH dehydrogenase 1 beta subcomplex 6, Mitochondrial peroxiredoxin-5, (PRDX5), Apolipoprotein A-I (APOA1), Mitochondrial Succinyl-CoA ligase [ADP-forming] subunit beta (SUCLA2), Acrosin-binding protein, Ropporin-1A, and Spermadhesin AWN that were enriched after-capacitation (>3-fold) by 2-DE and ESI-MS/MS. SUCLA2 and PDHB are involved in the tricarboxylic acid cycle, whereas PHGPx and PRDX5 are involved in glutathione metabolism. SUCLA2, APOA1 and PDHB mediate adipocytokine signaling and insulin action. The differentially expressed proteins following capacitation are putatively related to sperm functions, such as ROS and energy metabolism, motility, hyperactivation, the acrosome reaction, and sperm-egg interaction.

**Conclusion:**

The results from this study elucidate the proteins involved in capacitation, which may aid in the design of biomarkers that can be used to predict boar sperm quality.

## Background

Ejaculated spermatozoa undergo marked structural and biochemical changes within the female reproductive tract before fertilization. Although spermatozoa are motile and morphologically normal after ejaculation, they are unable to fertilize an oocyte. Subsequently, spermatozoa are exposed to a new environment where numerous chemicals in the female genital track trigger a cascade of metabolic and structural alterations associated with changes in membrane fluidity, intracellular bicarbonate and calcium levels, cAMP, PKA activity, and tyrosine phosphorylation of proteins [1–10]. This time-dependent acquisition of fertilizing competence has been termed “capacitation” [11, 12]. Proteomic studies have been conducted to elucidate the molecular mechanisms underlying capacitation for humans [13], mice [14], hamsters [15], boars [16], and bulls [17]. In most cases, these studies identified specific set of proteins and tyrosine-phosphorylated proteins that are involved in capacitation [13–17]. Mature spermatozoa are unable of transcription, translation, and protein synthesis [18]. However, a new viewpoint has been presented that ejaculated spermatozoa are capable of utilizing mRNA transcripts for protein translation during their functional maturation [19]. Simultaneously, it is well accepted that spermatozoa acquire their functionality via post-translational protein modifications such as phosphorylation [20, 21]. It has been demonstrated that freeze-thawing of human spermatozoa results in differential expression of twenty-seven proteins compare to their fresh ejaculate [20]. This study suggested that cryopreservation may be induced spermatozoa dysfunction due to protein degradation and protein phosphorylation [20, 21]. Therefore, it is a matter of paramount importance to detect a set of differentially expressed proteins associated with capacitation as well as other functional state of spermatozoa.

Successful fertilization requires that spermatozoa complete to capacitate at right time both *in vitro* and *in vivo*. Therefore, measuring the fraction of a sperm population that is able to capacitated will possibly be an excellent criteria to measure semen quality. Literature demonstrated that the prediction of male fertility of mammals still depends on conventional sperm analysis, such as sperm morphology [22–24], motility [25–27], and sperm penetration assays [28, 29], and their clinical value has been disputed [30]. Therefore, the accurate and broadly applicable methods for semen assessment might help to analyze male fertility.

Recent advances through performing two-dimensional electrophoresis (2-DE) for the separation of proteins and mass spectrometry (MS) for peptide sequencing have facilitated protein identification, leading to the rapid expansion of sperm proteomic research. A previous study in our laboratory established a suitable *in vitro* assay of male fertility for performing fertility-related proteomics of bull spermatozoa [31]. Therefore, the present study employed proteomic outlining of boar spermatozoa following capacitation in order to elucidate this extremely important event. A comprehensive and comparative proteomic study was carried out to explore the changes in protein expression (>3-fold) during capacitation. Sperm motility (%), motion kinematics, capacitation status, and tyrosine phosphorylation were analyzed using combined computer-assisted sperm analysis (CASA), Hoechst 33258/chlortetracycline fluorescence assessment (H33258/CTC), and Western blotting, respectively. Next, the 2-DE results were confirmed using Western blotting and immunocytochemistry. Finally, related signaling pathways were constructed based on the differentially expressed proteins.

## Results

### Sperm motility, motion kinematics, capacitation status, and tyrosine phosphorylation

**Figure 1:**
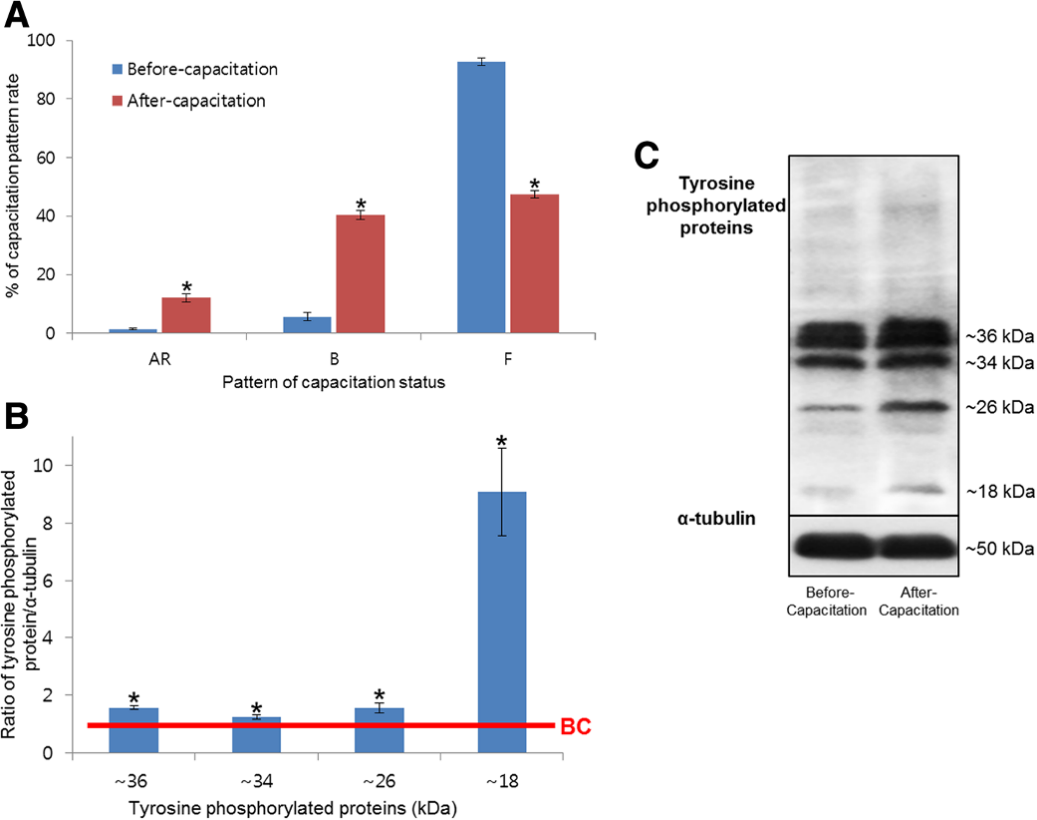
**Capacitation status and tyrosine phosphorylation of spermatozoa following incubation in capacitation media. (A)** Changes in capacitation pattern. Data represent mean ± SEM, n =3, *P <0.05. **(B)** Ratios of tyrosine phosphorylated proteins (optical density [OD] × mm)/α-tubulin (OD × mm). Data represent mean ± SEM, n =3, *P <0.05. The red line indicates the landmark of before-capacitation (BC). **(C)** Tyrosine-phosphorylated proteins were probed with anti-Phosphotyrosine (4G10) antibody; lane 1: before capacitation; lane 2: after capacitation.

To measure the motility parameters of before- and after-capacitation spermatozoa, we performed CASA technique as described in the Methods. A variety of motion parameters, including hyperactivated motility (HYP), curvilinear velocity (VCL), and mean amplitude of head lateral displacement (ALH) were significantly increased in after-capacitation compare to before-capacitation spermatozoa (*P* < 0.05, Table 1). However, straight-line velocity (VSL) was significantly decreased in after-capacitation (*P* < 0.05, Table 1). In present study, the dual staining method was performed to evaluate the changes in capacitation status both before- and after-capacitation spermatozoa. The acrosome reacted (AR) and capacitated (B) patterns were significantly increased in after-capacitation (*P* < 0.05), while the non-capacitated (F) pattern was significantly decreased in after-capacitation spermatozoa (*P* < 0.05). It has been demonstrated that capacitation of mammalian spermatozoa are associated to the activation of a cAMP/PKA-dependent signaling pathways followed by up-regulation of protein tyrosine phosphorylation [8, 9]. Therefore, next we measured the levels of tyrosine phosphorylation in both groups of spermatozoa. Four different tyrosine phosphorylated protein bands (approximately 18, 26, 34, and 36 kDa) were significantly increased in after-capacitation (*P* < 0.05, Figure 1) compare to before-capacitation spermatozoa.

**Table 1:** **Sperm motility and motion kinematics following capacitation**

Sperm motility and motion kinematics	Before-capacitation	After-capacitation
MOT (%)	85.33 ± 2.80	87.42 ± 1.77
HYP (%)	5.76 ± 1.42	15.89 ± 2.26*
VCL (μm/s)	119.56 ± 2.22	147.28 ± 5.04*
VSL (μm/s)	75.87 ± 0.97	64.85 ± 1.00*
VAP (μm/s)	81.06 ± 2.48	81.57 ± 2.51
ALH (μm)	5.9 ± 0.19	6.97 ± 0.24*

Sperm motility and motion kinematics are presented as mean ± SEM, n =3, *P <0.05. MOT = motility (%); HYP = hyperactivated sperm (%); VCL = curvilinear velocity (μm/s); VSL = straight-line velocity (μm/s); VAP = average path velocity (μm/s); ALH = mean amplitude of head lateral displacement (μm).

### Proteomic analysis and identification of capacitation proteins

**Figure 2:**
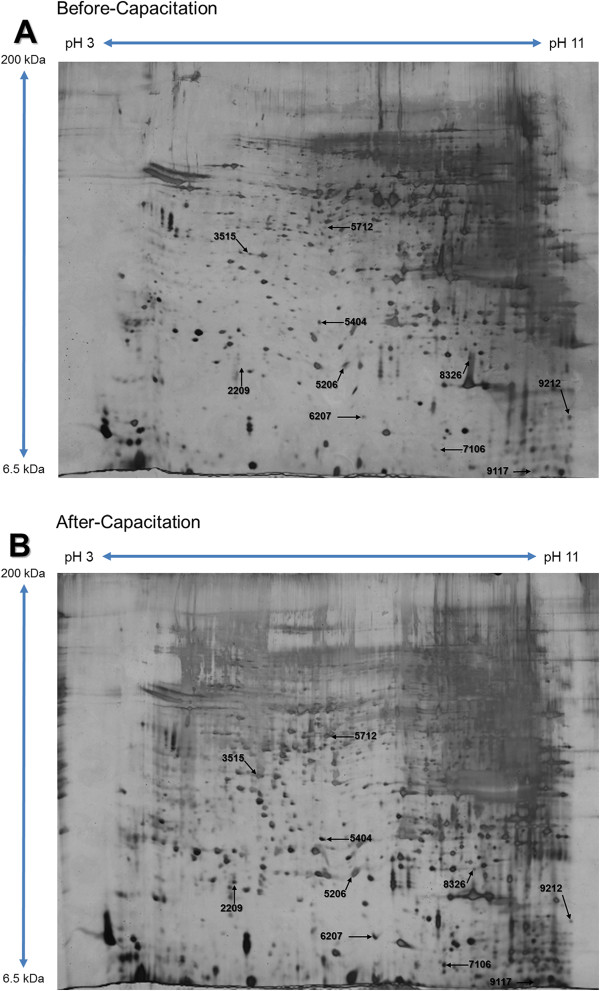
**Separation of proteins by 2-DE.** 2-DE gels were stained with silver nitrate and analyzed using PDQuest 8.0 software. **(A)** Protein spots from before-capacitation spermatozoa. **(B)** Protein spots from after-capacitation spermatozoa.

**Figure 3:**
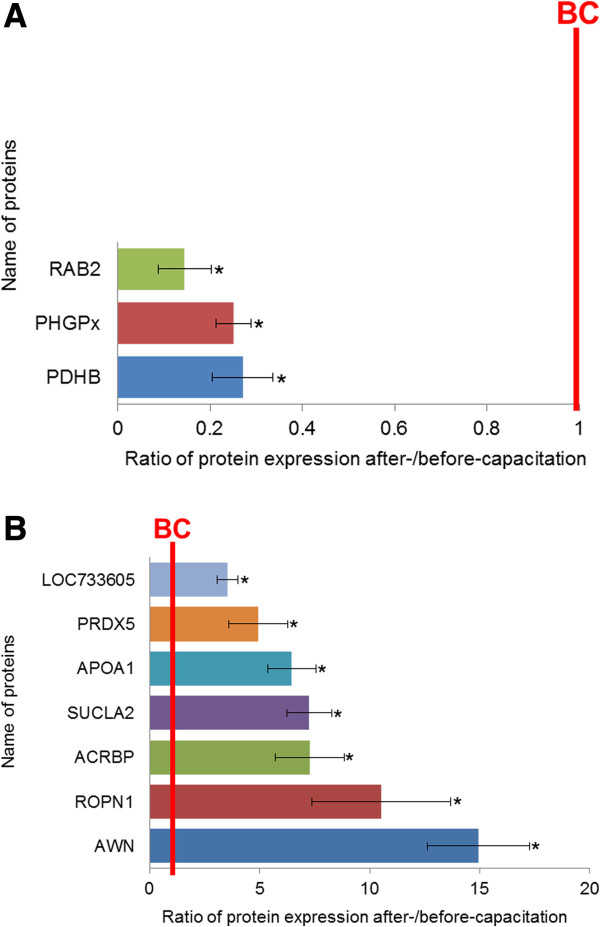
**Comparison of proteins from before- and after-capacitation spermatozoa.** Differentially expressed (>3-fold) proteins were determined by comparing before- and after-capacitation spermatozoa (^*^ *P* <0.05). The line indicates the landmark of before-capacitation (BC). **(A)** Three proteins were significantly decreased after capacitation. **(B)** Seven proteins were significantly increased after capacitation. The data represent the mean ± SEM, n =3.

A total of 224 protein spots were detected, and 10 spots showed significantly different expression (>3-fold difference; *P* < 0.05) between the before- and after-capacitation spermatozoa (Figure 2). Among them, 3 spots were enriched in the before-capacitation group, while 7 spots were enriched in the after-capacitation group (Figure 3). The differentially expressed spots (>3-fold) were identified by an MS/MS ion search using MASCOT software (Matrix Science). Notably, the 3 spots in the before-capacitation group included the Ras-related protein Rab-2 (RAB2), Phospholipid hydroperoxide glutathione peroxidase (PHGPx), and pyruvate dehydrogenase E1 component subunit beta, mitochondrial (PDHB). On the other hand, the 7 highly expressed spots in the after-capacitation group were NADH dehydrogenase 1 beta subcomplex 6 (LOC733605), Peroxiredoxin-5, mitochondrial (PRDX5), Apolipoprotein A-I (APOA1), Succinyl-CoA ligase [ADP-forming] subunit beta, mitochondrial (SUCLA2), Acrosin-binding protein (ACRBP), ropporin-1A (ROPN1), and spermadhesin AWN (AWN) (Table 2).

**Table 2:** **Differentially expressed (>3-fold) proteins identified by ESI-MS/MS**

Spot no.	gi no.	Symbol	Protein description	Peptide sequence	Mascot score ^*^
2209	gi|298160982	ROPN1	Ropporin-1A	R.LIIHADELAQMWK.V	105
				R.VALSNWAELTPELLK.I	
				R.MLNYIEQEVIGPDGLIK.V	
3515	gi|346986351	PDHB	Pyruvate dehydrogenase E1 component subunit beta, mitochondrial	R.IMEGPAFNFLDAPAVR.V	245
				K.TYYMSGGLQSVPIVFR.G	
				K.TTHLITVEGGWPQFGIGAEICAR.I	
5206	gi|464526	RAB2	Ras-related protein Rab-2	R.GAAGALLVYDITR.R	248
				R.DTFNHLTTWLEDAR.Q	
				R.FQPVHDLTIGVEFGAR.M	
5404	gi|89153	APOA1	Apolipoprotein A-I	R.DYVAQFEASALGK.H	34
5712	gi|21263966	SUCLA2	Succinyl-CoA ligase [ADP-forming] subunit beta, mitochondrial	K.LHGGTPANFLDVGGGATVHQVTEAFK.L	44
6207	gi|47523086	PRDX5	Peroxiredoxin-5, mitochondrial	R.LLADPTGAFGK.E	150
				R.FSMVIEDGIVK.S	
				K.VGDAIPSVVVFEGEPEKK.V	
7106	gi|75052483	ACRBP	Acrosin-binding protein	R.FYGLDLYGGLR.M	125
				R.VASWLQTEFLSFQDGDFPTK.I	
8326	gi|13195731	GPX4	Phospholipid hydroperoxide glutathione peroxidase	K.TEVNYTQLVDLHAR.Y	109
				R.QEPGSDAEIKEFAAGYNVK.F	
9117	gi|66990208	AWN	Spermadhesin AWN	K.EYVELLDGPPGSEIIGK.I	173
				R.ASPFHIYYYADPEGPLPFPYFER.Q	
9212	gi|113205666	LOC733605	NADH dehydrogenase 1 beta subcomplex 6	R.IFPGDTILETGEVIPLMK.E	48

*MASCOT score is -10 log (*P*), where *P* is the probability that the observed match is a random event. Individual scores >30 indicate identity or extensive homology (*P* < 0.05).

### Protein confirmation by western blotting and immunofluorescence

**Figure 4:**
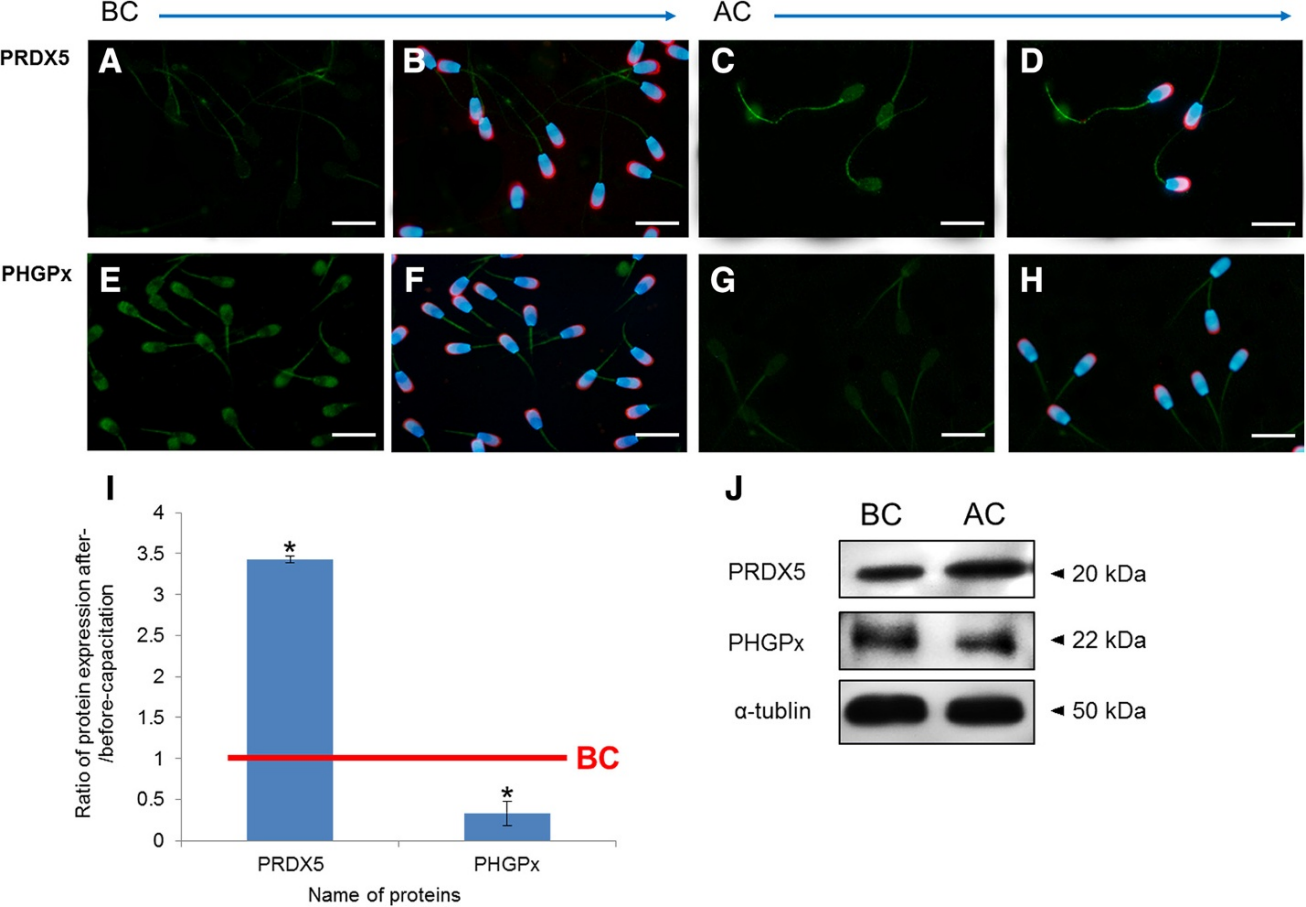
**Localization and expression of PRDX5 and PHGPx before- and after-capacitation in porcine spermatozoa. (A and E)** Images of PRDX5 and PHGPx before capacitation (green). **(B and F)** Merged image of the nucleus (DAPI, blue) and acrosome (lectin PNA, red) with PRDX5 and PHGPx before-capacitation, respectively (green). **(C and G)** Images of PRDX5 and PHGPx after-capacitation (green). **(D and H)** Merged image of nucleus (DAPI, blue) and acrosome (lectin PNA, red) with PRDX5 and PHGPx after-capacitation, respectively (green). Images were obtained using a Nikon TS-1000 microscope and NIS Elements image software (Nikon, Japan). Bar =10 μm. **(I)** Ratios of PRDX5 and PHGPx [optical density (OD x mm)/α-tubulin (OD x mm)] before- and after-capacitation. Data represent mean ± SEM, n =3. Proteins expression ratios with superscripts were significantly different (*P < 0.05). **(J)** PRDX5 and PHGPx were probed with anti-PRDX5 and anti-PHGPx antibody.

To validate the 2-DE results, the differentially expressed proteins were further examined by Western blotting analysis by using commercially available antibodies. In after-capacitation spermatozoa, PRDX5 and PHGPx were detected at ~20 and 22 kDa, respectively. The density of PRDX5 increased (*P* < 0.05), while the density of PHGPx was decreased (*P* < 0.05) following capacitation. Additionally, these proteins were detected by immunofluorescence using the corresponding antibodies along with lectin PNA (to detect the acrosomal region) and DAPI (to detect the nucleus) in spermatozoa following capacitation. PRDX5 was detected in the midpiece, and PHGPx was detected in the acrosome and midpiece. These protein expression patterns were similar to the expression patterns detected by Western blotting (Figure 4).

### Signaling pathway

**Figure 5:**
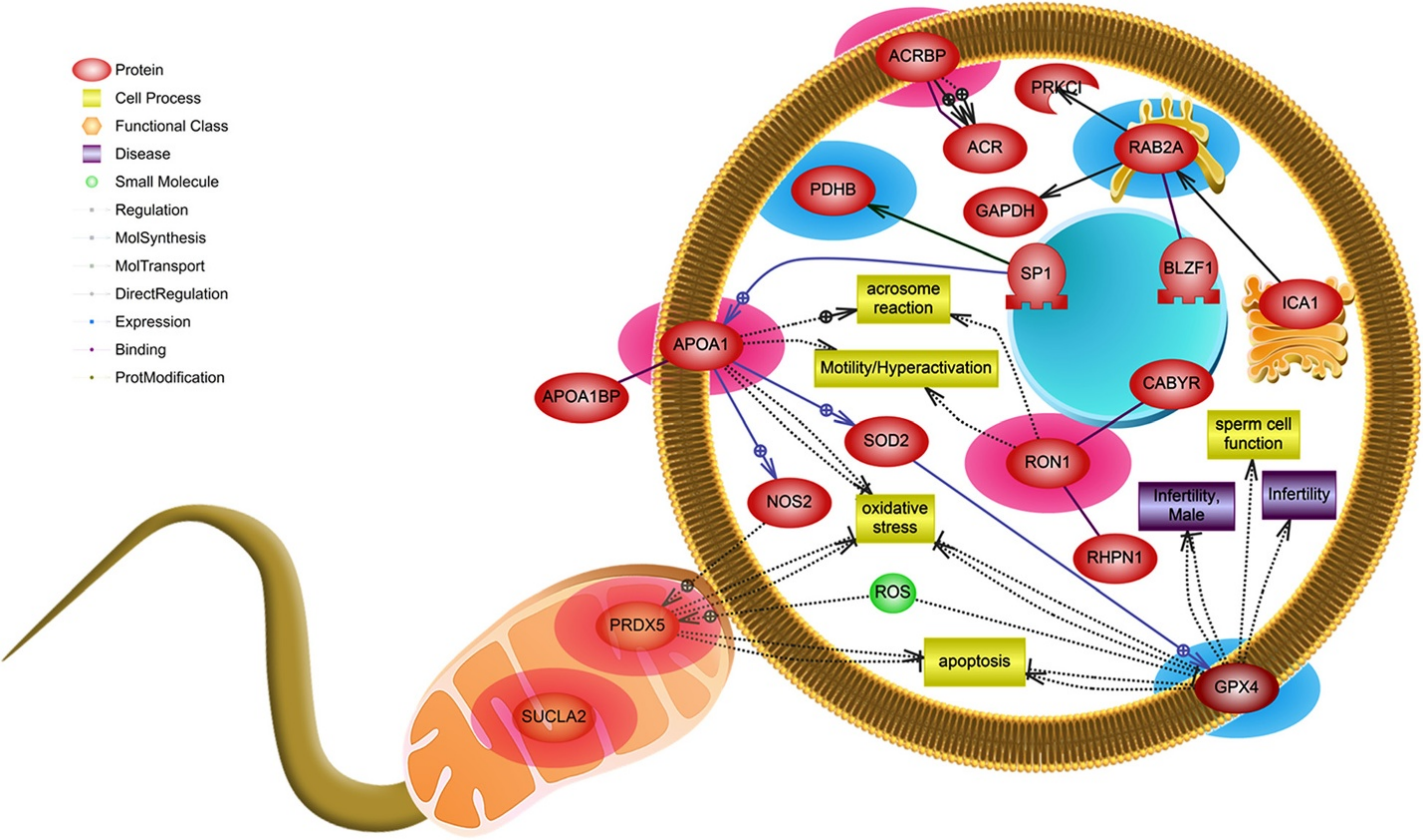
**Signaling pathways associated with capacitation-related proteins.** The pathway was drawn using Pathway Studio 9.0 after a database search in PubMed. Red denotes the proteins that were abundantly expressed after-capacitation, while blue denotes proteins that were abundantly expressed before-capacitation. At least 8 proteins were implicated in different sperm-specific process among the 10 proteins.

The gene name of each differentially expressed protein was confirmed by performing a database search, and the results were imported into Pathway Studio to identify their signaling pathways. Three pathways were significantly correlated with 5 of the proteins (Table 3, *P* < 0.05). SUCLA2 and PDHB were significantly correlated with the tricarboxylic acid cycle, whereas PHGPx and PRDX5 were significantly correlated with glutathione metabolism (Table 3, *P* < 0.05). SUCLA2, APOA1, and PDHB were correlated with adipocytokine signaling and insulin action (Table 3, *P* < 0.05). Figure 5 illustrates the cellular pathways regulated by the differentially expressed proteins in spermatozoa [18]. At least 8 proteins were implicated in different sperm-specific process. These proteins were putatively related to sperm functions, such as ROS and energy metabolism, motility, hyperactivation, the acrosome reaction, and male fertility.

**Table 3:** **Signaling pathways associated with differentially expressed proteins as identified by Pathway Studio**

Signaling pathways	Overlapping entities	***P*** -value
**Metabolic Pathways**		
Tricarboxylic acid cycle	SUCLA2, PDHB	0.002
Glutathione metabolism	PHGPx, PRDX5	0.011
**Cell Signaling Pathways**		
Adipocytokine signaling	SUCLA2, APOA1, PDHB	0.008
Insulin action	SUCLA2, APOA1, PDHB	0.012

Differentially expressed proteins were entered into Pathway Studio to identify the corresponding signaling pathways that potentially regulate capacitation. Among the differentially expressed proteins (>3-fold) of before- and after-capacitation, at least 5 exhibited regulatory roles in single or more pathways simultaneously (*P* < 0.05).

## Discussion

Proteomic techniques have enabled the investigation of sperm-specific cellular processes. Such studies have been pivotal in identifying and designing valid biomarkers of male fertility [31, 32]. Spermatozoa must undergo capacitation before fertilizing an oocyte [9, 10], and the process is associated with changes in protein content. It is generally believed that spermatozoa are unable to transcription, translation, and protein synthesis [33, 34]. However, reports also exist spermatozoa are capable of synthesis new proteins [35]. Therefore, proteomic profiling of boar spermatozoa following capacitation may elucidate the proteins involved in capacitation.

Capacitation is an important pre-requisite to fertilization. During this process, tyrosine phosphorylation occurs in spermatozoa, which triggers a change in motion kinematics and morphology [1–5]. In the present study, capacitation was induced by heparin and was confirmed by measuring changes in tyrosine phosphorylation, sperm motility, motion kinematics, and CTC patterns. As a result of capacitation, there was a significant increase in tyrosine-phosphorylated proteins, HYP, VCL, ALH, and capacitation status (*P* <0.05, Table 1 and Figure 1). In addition, VSL was a significantly decreased in after-capacitation (*P* <0.05, Table 1). These results indicate that we achieved optimum capacitation *in vitro* in boar spermatozoa.

To identify marked changes in protein expression (>3-fold), spermatozoa were separated by Percoll and analyzed by 2-DE in before- and after-capacitation (Table 2 and Figure 2). Three proteins were abundant in before-capacitation, and 7 were abundant in after-capacitation (Figure 3). Finally, we used Pathway Studio to search for the identified proteins and construct signaling pathways involved in the capacitation process. To validate the 2-DE results, Western blotting and immunofluorescence were performed with commercially available antibodies to detect the differentially expressed proteins.

RAB2, PHGPx, and PDHB were decreased after capacitation (Figure 3A). RAB2A and RAB2B are 2 subgroups of RAB2 proteins [36]. Members of the RAB family of proteins play a critical role in regulating vesicular transport and membrane fusion and are localized to the acrosomal membrane during acrosome formation in spermatozoa [36–38]. Capacitated spermatozoa undergo the acrosome reaction, a process of acrosomal exocytosis [39, 40]. Therefore decreased expression of RAB2 after-capacitation suggests that RAB2 is involved in structural modification of the acrosome to induce acrosomal exocytosis following capacitation. On the other hand, PHGPx contributes to cross-linking in mitochondrial capsules of mammalian spermatozoa and provides structural stability [41, 42]. Therefore, similar to RAB2, the decrease PHGPx in spermatozoa after capacitation (Figures 3A, 4I, and J) might promote acrosomal exocytosis. Foresta et al. reported that PHGPx was important for fertility [43]. They also reported that its over-expression increased the resistance of mouse NSC-34 motor neuron-like cells, which is thought to induce mitochondrial abnormalities in patients with amyotrophic lateral sclerosis. In the present study, we identified PHGPx in the head and tail of spermatozoa (Figure 4) and predicted its involvement in regulating glutathione metabolism based on an analysis in Pathway Studio (Table 3). These results indicated that before-capacitation spermatozoa metabolize glutathione, which may facilitate capacitation in spermatozoa by promoting the acrosome reaction, fertilization, and other events.

Another protein that decreased after capacitation was PDHB (Figure 3A). PDHB is one of 7 subunits of the pyruvate dehydrogenase complex (PDH) family, which converts pyruvate to acetyl-CoA during aerobic oxidation of glucose [44–46]. PDHB is a phospho-tyrosine protein involved in capacitation in human spermatozoa [47]. In the present study, we used Pathway Studio to identify PDHB’s potential involvement in regulating the tricarboxylic acid cycle, adipocytokine, and insulin action (Table 3). Together with its localization in sperm flagella [48], these finding provide further insight into PDHB’s involvement in energy production and metabolism in order to control sperm motility and hyperactivation during capacitation.

The proteins that increased (>3-fold) in after-capacitation included LOC733605, PRDX5, APOA1, SUCLA2, ACRBP, ROPN1, and AWN (Figure 3B). PRDX5 is a thioredoxin peroxidase that protects spermatozoa from oxidative stress [49, 50]. Recently, O’Flaherty and de Souza reported that PRDX5 was localized to the acrosome, post-acrosome and midpiece of spermatozoa [51]. Its localization to multiple sites indicates its potential involvement in different physiological processes responsible for fertilization in the spermatozoon. In the present study, we identified PRDX5 in head and midpiece of spermatozoa (Figure 4). Its increased expression after capacitation (Figures 3B, 4I and J) and its localization suggest that PRDX5 plays an important role in sperm-oocyte binding as well as regulating energy production by mitochondria in the midpiece. A similar result was also reported in an earlier study in boar spermatozoa [52].

APOA1 has been reported to induce cholesterol efflux in spermatozoa [53, 54]. Cholesterol efflux from the sperm membrane regulates multiple signaling cascades responsible for motility, hyperactivation, and capacitation [55]. Thérien et al. reported that APOA1 is part of high-density lipoprotein (HDL) and triggers the acrosome reaction [56]. The APOA1 is known as a seminal plasma protein that affects sperm-oocyte binding and ultimately male fertility. In present study, APOA1 was increased after capacitation. It is plausible to suggest that APOA1 come from fetal bovine serum used in capacitation media. However, we identified this protein in mature spermatozoa where the seminal plasma had removed completely by discontinuous Percoll gradients [16, 31, 52, 57]. Therefore, our study provides evidence that APOA1 might also be present in spermatozoa and play an essential role in fertilization.

In the present study, we identified increased expression of ACRBP in after-capacitation. ACRBP regulates the release of acrosin from the acrosome of spermatozoa [58]. Acrosin is the major proteinase that lyses the zona pellucida and facilitates the penetration of the sperm through the innermost glycoprotein layers of the ovum. Interestingly, recent study suggested that ACRBP can be used as marker to predict boar sperm freezability [59]. Therefore, the capacitation-associated increase in ACRBP plays an important role in the control of male fertility. Another capacitation-induced protein is ROPN1. Fujita et al. reported that ROPN1 is localized to the sperm flagella [60]. Another study reported decreased expression of ROPN1 in the spermatozoa of patients with low sperm motility [61]. Therefore, we hypothesize that ROPN1 contributes to sperm hyperactivation. Indeed, sperm hyperactivation facilitate the release from oviductal storage and propels them into the oviductal lumen and matrix of the cumulus oophorus during fertilization.

NADH dehydrogenase and succinyl-CoA ligase are located in sites I and II of the mitochondrial electron transport chain, respectively. Therefore, these enzymes affect motility and hyperactivation of human spermatozoa [62]. NADH dehydrogenase and succinyl-CoA ligase were identified as substrates of protein kinase A (PKA). In the present study, LOC733605 and SUCLA2 were increased in after-capacitation (Figure 3B). In addition, we demonstrated the potential involvement of SUCLA2 in the tricarboxylic acid cycle, adipocytokine signaling, and insulin action by Pathway Studio (Table 3). Therefore, spermatozoa may participate in various metabolic and cell signaling pathways after capacitation that affects the subsequent acrosome reaction and fertilization.

The spermadhesin proteins are constituents of boar seminal plasma, attach to the sperm acrosome thereby assist sperm-egg interaction [63]. Five spermadhesin proteins were identified in the boar semen, such as PSP-I, PSP-II, AQN-1, AQN-3, and AWN [64]. A review of literature demonstrated that spermadhesin AWN regulates the phospholipid-binding activity in spermatozoa, thus promoting the capacitation and the acrosomal stabilization [65]. Therefore, high levels of AWN after capacitation (Figure 3B) might represent the potentiality of the capacitated spermatozoa to bind with eggs zona pellucida during the initial stages of the sperm-egg interaction for fertilization. Since, only capacitated spermatozoa are able to undergo the acrosome reaction, binding to the zona pellucida, and fusion to the oocyte membrane [66]. In contrast, Dostàlovà et al. [65] reported that the spermadhesin content in boar spermatozoa had been lost during capacitation. This conclusion did not entirely support the finding of present study. Therefore, further studies are required to investigate the role of AWN in capacitation, the acrosome reaction, fertilization, and beyond.

## Conclusion

In the present study, we performed comprehensive proteomic profiling of boar spermatozoa under capacitation conditions. We identified proteins involved in capacitation and their signaling pathways. To the best of our knowledge, this is the first study to identify 10 proteins that undergo dramatic changes in expression (>3-fold) during capacitation in boar spermatozoa. Additionally, Pathway Studio was used to identify at least 5 proteins implicated signaling pathways (Table 3) and to illustrate the cellular pathways regulated by the identified proteins in spermatozoa (Figure 5). However, a few of the identified proteins have unknown functions. In addition, some proteins exhibited various functions, and although they have diverse roles in the whole organism, their specific functions in spermatozoa are unknown. Therefore, further studies must identify the functions of these proteins in sperm cells. Likewise, these candidate markers might be useful for designing diagnostic tools to evaluate and/or predict fertility-related diseases and male infertility during capacitation.

## Methods

### Sample preparation

Semen samples were collected from 12 individual Landrace males (Sunjin Co., Danyang, Korea) with normal fertility (pregnancy rate, 90% ±1.44; average litter size, 10.75 ± 0.39), and samples were divided randomly into 3 groups for experimental replication (n = 4). To avoid individual male factors, each group’s semen samples were mixed together. Pooled samples were washed at 500 × *g* for 20 min with a discontinuous (70% [v/v] and 35% [v/v]) Percoll gradient (Sigma, St Louis, MO, USA) to remove seminal plasma and dead spermatozoa [16]. Then, the washed samples were divided into 2 groups: before-capacitation and after-capacitation spermatozoa. For the after-capacitation samples, further incubation was performed with modified tissue culture media (mTCM) 199 (containing 10% fetal bovine serum [v/v], 0.91 mM sodium pyruvate, 3.05 mM D-glucose, 2.92 mM calcium lactate, 2.2 g/L sodium bicarbonate and 10 μg/mL heparin) (Sigma, St Louis, MO, USA) for 30 min at 37°C under an atmosphere of 5% CO_2_ in air [26, 27, 66]. All procedures were performed according to guidelines for the ethical treatment of animals and approved by Institutional Animal Care and Use Committee of Chung-Ang University.

### Computer-assisted sperm analysis

To analyze motility and motion kinematics of before-capacitation sample, the sample was pre-incubated with mTCM 199 (without 10% fetal bovine serum [v/v] and 10 μg/mL heparin) for 10 min at 37°C under an atmosphere of 5% CO_2_ in air. The same parameters of after-capacitation sample were analyzed following 30 min incubation of the sample with capacitation media (mention earlier). A computer-assisted sperm analysis (CASA) system (sperm analysis imaging system version (SAIS)-PLUS 10.1; Medical Supply, Seoul, Korea) was used to analyze sperm motility (%) and motion kinematics. Briefly, 10 μL of sample was placed in a Makler chamber (Makler, Haifa, Israel). The filled chamber was placed on a stage preheated to 37°C. Using a 10 × objective in-phase contrast mode, the image was relayed, digitized, and analyzed by SAIS software. The movement of at least 250 sperm cells was recorded for each sample from more than five randomly selected fields per replicate.

### H33258/CTC assessment of capacitation status

Capacitation status was determined by the dual staining method (H33258/CTC) described previous [9, 10, 67] Briefly, 135 μL of treated spermatozoa were added to 15 μL of H33258 solution (10 μg H33258/mL Dulbecco’s phosphate buffered saline (DPBS) and incubated for 2 min at room temperature (RT). Excess dye was removed by layering the mixture over 250 μL of 2% (w/v) polyvinylpyrrolidone in DPBS. The supernatant was discarded after centrifuging at 100 × g for 2.5 min. The pellet was resuspended in 100 μL of DPBS; 100 μl of a chlortetracycline fluorescence (CTC) solution (750 mM CTC in 5 μL buffer composed of 20 mM Tris, 130 mM NaCl, and 5 mM cysteine, pH 7.4). Capacitation status was observed with a Microphot-FXA microscope (Nikon) under epifluorescent illumination using ultraviolet BP 340–380/LP 425 and BP 450–490/LP 515 excitation/emission filters for H33258 and CTC, respectively. The patterns of capacitation status in the spermatozoa were classified as live non-capacitated (F, bright green fluorescence distributed uniformly over entire sperm head, with or without a stronger fluorescent line at the equatorial segment), live capacitated (B, green fluorescence over the acrosomal region and a dark post-acrosomal region), or live acrosome reacted (AR, sperm showing a mottled green fluorescence over the head, green fluorescence only in the post acrosomal region, or no fluorescence over the head) [9, 10]. Two slides per sample were evaluated with at least 400 spermatozoa per slide.

### 2DE and gel-image analysis

To extract proteins from the spermatozoa, 50 × 10^6^ spermatozoa were incubated in rehydration buffer containing 7 M urea (Sigma, St Louis, MO, USA), 2 M thiourea (Sigma, St Louis, MO, USA), 4% (w/v) CHAPS (USB, Cleveland, OH, USA), 0.05% (v/v) Triton X-100 (Sigma, St Louis, MO, USA), 1% (w/v) octyl β-D-glucopyranoside, 24 μM PMSF (Sigma, St Louis, MO, USA), 1% (w/v) DTT (Sigma, St Louis, MO, USA), 0.5% (v/v) IPG Buffer, and 0.002% (w/v) bromophenol blue at 4°C for 1 h. Then, 250 μg of solubilized protein from the sperm cells in 450 μL of rehydration buffer was placed in a rehydration tray with 24 cm-long NL Immobiline DryStrips (pH 3–11; Amersham, Piscataway, NJ, USA) for 12 h at 4°C. First dimension electrophoresis was performed using an IPGphor IEF device and then the strips were focused at 100 V for 1 h, 200 V for 1 h, 500 V for 1 h, 1,000 V for 1 h, 5,000 V for 1.5 h, 10 8,000 V for 1.5 h, and 8,000-90,000 Vhr. After iso-electrofocusing, the strips were equilibrated a second After iso-electrofocusing, the strips were equilibrated with equilibration buffer A containing 6 M urea, 75 mM Tris–HCl (pH 8.8), 30% (v/v) glycerol, 2% (w/v) SDS, 0.002% (w/v) bromophenol blue, and 2% (w/v) DTT for 15 min at RT. The strips were equilibrated for a second time with equilibration buffer B (equilibration buffer A with 2.5% [w/v] iodoacetamide [Sigma] but without DTT for 15 min at RT. Next, 2-DE was carried out with 12.5% (w/v) SDS-PAGE gels with the strips at 100 V for 1 h and 500 V until the bromophenol blue front began to migrate off the gels. The gels were silver-stained for image analysis according to the manufacturer’s instructions (Amersham, Piscataway, NJ, USA). The gels were then scanned using a high-resolution GS-800 calibrated scanner (Bio-Rad, Hercules, CA, USA). Detected spots were matched and analyzed by comparing the gels from spermatozoa before- and after-capacitation using PDQuest 8.0 software (Bio-Rad, Hercules, CA, USA). The gel from before-capacitation spermatozoa was used as a control. Finally, the density of the spots was calculated and normalized as the ratio of the spot on the after-capacitation gel to that on the before-capacitation gel.

### In-gel digestion

The proteins were subjected to in-gel trypsin digestion. Excised gel spots were destained with 100 μl of destaining solution (30 mM potassium ferricyanide and 100 mM sodium thiosulfate) with shaking for 5 min. After removing the solution, the gel spots were incubated with 200 mM ammonium bicarbonate for 20 min. The gel pieces were dehydrated with 100 μL of acetonitrile and dried in a vacuum centrifuge. The above procedure was repeated 3 times. The dried gel pieces were rehydrated with 20 μL of 50 mM ammonium bicarbonate containing 0.2 μg modified trypsin (Promega, Madison, WI, USA) for 45 min on ice. After removing the solution, 30 μL of 50 mM ammonium bicarbonate was added. The digestion was performed overnight at 37°C. The peptide solution was desalted using a C18 nano column (homemade, Waters Corp, Milford, MA, USA).

### Desalting and concentration

Custom-made chromatographic columns were used for desalting and concentrating the peptide mixture prior to MS analysis. A column consisting of 100–300 nL of Poros reverse phase R2 material (20–30 μm bead size, Perseptive Biosystems, Framingham, MA, USA) was packed in a constricted GELoader tip (Eppendorf, Hamburg, Germany). A 10 mL syringe was used to force liquid through the column by applying gentle air pressure. Thirty microliters of the peptide mixture from the digestion supernatant was diluted in 30 μL of 5% formic acid, loaded onto the column, and washed with 30 μL of 5% formic acid. For MS/MS analyses, the peptides were eluted with 1.5 μL of 50% methanol/49% H_2_O/1% formic acid directly into a pre-coated borosilicate nano-electrospray needle (New Objective, Woburn, MA, USA).

### ESI-MS/MS

Proteins generated by in-gel digestion were subjected to MS/MS using a nano-ESI on a Q-TOF2 mass spectrometer (AB Sciex Instruments, Framingham, MA, USA). The source temperature was RT. A potential of 1 kV was applied to the pre-coated borosilicate nano-electrospray needles (New Objective, Woburn, MA, USA) in the ion source, combined with a nitrogen back-pressure of 0–5 psi to produce a stable flow rate (10–30 nL/min). The cone voltage was 40 V. A quadrupole analyzer was used to select precursor ions for fragmentation in the hexapole collision cell. The collision gas was argon at a pressure of 6–7 × 10^-5^ mbar and the collision energy was 25–40 V. Product ions were analyzed using an orthogonal TOF analyzer that was fitted with a reflector, which was a micro-channel plate detector, and a time-to-digital converter. The data were processed using a peptide sequencing system.

### Database search

A MS/MS ion search was assigned as the ion search option in MASCOT software (Matrix Science, Boston, MA, USA). Peptide fragment files were obtained from the peptide peaks in ESI-MS by ESI-MS/MS. Trypsin was selected as the enzyme with one potentially missed cleavage site. ESI-QTOF was selected as the instrument type. The peptide fragment files were searched within the database using the Mascot search engine (Matrix Science, Boston, MA, USA), and results were limited to *Sus scrofa* taxonomy. Oxidized methionine was set as a variable modification, and carbamidomethylated cysteine was set as a fixed modification. The mass tolerance was set at ±1 and ±0.6 Da for the peptides and fragments, respectively. High-scoring was defined as those above the default significance threshold in MASCOT (*P* < 0.05, peptide score >30).

### Western blotting

To evaluate the capacitation status, tyrosine phosphorylated proteins were detected with an anti-Phosphotyrosine (4G10) antibody. In order to confirm the 2-DE results, Western blotting was performed with anti-phospholipid hydroperoxide glutathione peroxidase (PHGPx) and anti-peroxiredoxin-5, mitochondrial (PRDX5) antibodies to quantify 3 individual boar spermatozoa before- and after-capacitation. Western blotting was performed as described previously with modification [9, 10, 68]. The samples were washed twice with DPBS and centrifuged at 10,000 × *g* for 5 min. Afterwards, the pellets were re-suspended and incubated with sample buffer containing 5% 2-mercaptoethanol for 10 min at RT. After incubation, the insoluble fractions were separated by centrifugation at 10,000 × *g* for 10 min. The samples were subjected to SDS-PAGEs using a 12% mini-gel system (Amersham, Piscataway, NJ, USA), and the separated proteins were transferred to PVDF membranes (Amersham, Piscataway, NJ, USA). The membranes were blocked with 3% blocking agent (Amersham, Piscataway, NJ, USA) for 1 h at RT. Tyrosine phosphorylated proteins, PHGPx and PRDX5 proteins from before- and after-capacitation spermatozoa were immunodetected with an anti-Phosphotyrosine (4G10) mouse polyclonal antibody (Millipore, Darmstadt, Germany), anti-PHGPx rabbit polyclonal antibody (Abcam, Cambridge, UK), and anti-PRDX5 rabbit polyclonal antibody (Abcam, Cambridge, UK), respectively, that were diluted in 3% blocking agent (1 μg/ml) for 2 h at RT. Then, the membranes were incubated with an HRP conjugated goat anti-mouse IgG or anti-rabbit IgG (Abcam, Cambridge, UK) diluted in 3% blocking agent (1:5,000) for 1 h at RT. The membranes were washed 3 times with DPBS containing 0.1% Tween-20 (PBS-T). The proteins on the membranes were detected with an enhanced chemiluminescence (ECL) technique using ECL reagents. Proteins on membranes were stripped with membrane stripping solution (2% SDS, 100 mM mercaptoethanol and 62 mM Tris-cl) after detection. Then, α-tubulin was detected by incubation with a monoclonal anti-α-tubulin mouse antibody (Abcam, Cambridge, UK) diluted in 3% blocking agent (1:10,000) for 2 h at RT. Membranes were incubated with an HRP-conjugated goat anti-mouse IgG (Abcam, Cambridge, UK) diluted in 3% blocking agent (1:10,000) for 1 h at RT. The α-tubulin on the membranes was detected with an ECL technique using ECL reagents. All bands were scanned with a GS-800 calibrated imaging densitometer (Bio-Rad, Hercules, CA, USA) and analyzed with Quantity One software (Bio-Rad, Hercules, CA, USA). Finally, the signal intensity ratios of the bands were calculated for tyrosine phosphorylated proteins, PHGPx, and PRDX5 as compared with α-tubulin.

### Immunofluorescence assay

To confirm the cellular localization of PHGPx and PRDX5, immunocytochemistry was performed in before- and after-capacitation spermatozoa. Before- and after-capacitation sperm suspensions were placed on slides and then dried. The slides were fixed with 3.7% paraformaldehyde for 30 min at 4°C, washed with PBS-T, and blocked with blocking solution (5% BSA in PBS-T) for 1 h at 37°C. Sample were incubated with anti-PHGPx and anti-PRDX5 rabbit polyclonal antibodies (Amersham, Piscataway, NJ, USA) diluted in blocking solution (1:200) and lectin Peanut agglutinin (PNA) conjugated with Alexa Fluor 647 (Molecular Probes, Carlsbad, CA, USA) diluted in blocking solution (1:100) overnight at 4°C. Then, the slides were incubated for 2 h at RT with fluorescein isothiocyanate conjugated goat anti-rabbit IgG (Abcam, Cambridge, UK) diluted in blocking solution (1:200) for 2 h at RT. Spermatozoa were counterstained with DAPI. The immunofluorescence signals were visualized under × 600 magnifications with a Nikon TS-1000 microscope using NIS Elements image software (Nikon, Tyoko, Japan).

### Signaling pathway

Pathway Studio (v 9.0, Aridane Genomics, Rockville, MD, USA) was used to predict the biological functions and signaling pathways of the differentially expressed proteins. A list of identified proteins was entered into Pathway Studio in order to determine matching pathways for each protein. Metabolic pathways and cell signaling pathways were confirmed by the PubMed Medline hyperlink that was embedded in each node.

### Statistical analysis

The data were analyzed with SPSS (v. 18.0, Chicago, IL, USA). The student’s two-tailed *t*-test was used to compare the capacitation conditions after normality and variance homogeneity test. *P* values < 0.05 were considered statistically significant. All data are expressed as mean ± SEM. The probabilities of the signaling pathways were determined using the Fisher exact test (*P* < 0.05).
